# Early pregnancy GDF15 trajectories and their association with normal gestation, pregnancy loss, and hyperemesis gravidarum

**DOI:** 10.1016/j.ebiom.2026.106428

**Published:** 2026-08-06

**Authors:** Jens Skjoldan Svenningsen, Anne Ostenfeld, Jesper Friis Petersen, Mikkel Skjoldan Svenningsen, Thore Hillig, Matthew P. Gillum, Trisha J. Grevengoed, Ellen Christine Leth Løkkegaard

**Affiliations:** a Department of Biomedical Sciences, Faculty of Health and Medical Sciences, University of Copenhagen, Copenhagen, Denmark; b Department of Obstetrics and Gynecology, Copenhagen University Hospital, North Zealand, Hilleroed, Denmark; c Department of Clinical Biochemistry, Copenhagen University Hospital, North Zealand, Hilleroed, Denmark

**Keywords:** GDF15, Early pregnancy, Hyperemesis gravidarum, PUQE-24, Nausea and vomiting of pregnancy, Pregnancy biomarkers, Longitudinal cohort study, Early pregnancy loss

## Abstract

**Background:**

Growth differentiation factor 15 (GDF15) rises dramatically in early pregnancy and has been linked to nausea, vomiting, and hyperemesis gravidarum (HG). Significant challenges remain as GDF15 levels overlap between normal and complicated pregnancies, limiting its current clinical utility as a biomarker. Furthermore, early pregnancy GDF15 trajectories remain poorly characterised because most studies lack longitudinal sampling and rely on single or infrequent measurements.

**Methods:**

We analysed longitudinal serum GDF15 samples and Pregnancy Unique Quantification of Emesis 24 (PUQE-24) questionnaire scores from women enrolled in two early pregnancy clinical trials (PEP Cohort and VOMIT Study), using robust nonlinear statistical models to characterise trajectories. Analyses included uncomplicated (reference) pregnancies, early pregnancy loss (EPL), and HG.

**Findings:**

In normal pregnancies, GDF15 rose dramatically until week 7, and plateaued by week 9, with a sigmoidal model fit (P < 0.01). Pregnancies with female foetuses had approximately 19% higher GDF15 (P < 0.01), and the rise occurred one day later in male foetuses (P < 0.02). PUQE-24 scores in normal pregnancy peaked around week 8 and declined thereafter, aligning with the GDF15 plateau. In women with HG, GDF15 plateaued by the time of diagnosis, with higher levels compared to normal pregnancies in the early window (P < 0.05) but overlapping confidence intervals by week 8. EPL showed lower GDF15 levels and a slower rise, but also heterogeneity between types of EPL.

**Interpretation:**

GDF15 trajectories in early pregnancy diverge by outcome and foetal sex. These patterns clarify GDF15 dynamics in normal pregnancy and complications such as HG and EPL and define optimal sampling windows and tentative reference intervals. These findings support further evaluation of GDF15 as a biomarker to improve diagnosis and guide treatment.

**Funding:**

Independent Research Fund Denmark. The funder of the study had no role in study design, data collection, data analysis, data interpretation, or writing.

**Research in context**

**Evidence before this study**

We searched PubMed from database inception to December 2025 using the terms “GDF15” OR “growth differentiation factor 15” OR “Macrophage Inhibitory Cytokine-1” AND “pregnancy” OR “nausea” OR “vomiting” OR “hyperemesis gravidarum”. Eligible studies included observational cohorts, case control studies, genome-wide association studies, and mechanistic research on GDF15 and its receptor GFRAL–RET. Over the past decade, studies led by Marlena Fejzo and colleagues have defined the modern understanding of GDF15 in relation to pregnancy and hyperemesis gravidarum (HG), showing through genetic analyses that maternal variants near the GDF15 locus are the strongest known risk factors for HG, and demonstrating that maternal and foetal GDF15 levels together influence nausea and vomiting in pregnancy. Clinical studies have consistently found higher early pregnancy GDF15 concentrations in individuals with HG compared with normal pregnancies, although there is considerable overlap in absolute values. Most available studies rely on single or widely spaced sampling, leaving early pregnancy GDF15 trajectories insufficiently described.

**Added value of this study**

This study provides detailed longitudinal first trimester GDF15 measurements alongside repeated PUQE-24 scores in a large cohort of individuals with uncomplicated pregnancies, enabling the description of normal early pregnancy trajectories and a tentative reference interval. By comparing these trajectories with those of individuals suffering early pregnancy loss or HG, this study extends previous findings and demonstrates how early GDF15 dynamics diverge according to pregnancy outcome.

**Implications of all the available evidence**

Together with existing mechanistic, genetic, and clinical studies, these findings strengthen the evidence that GDF15 plays a central role in early pregnancy development of nausea, vomiting, and HG. By establishing typical first trimester GDF15 trajectories and comparing with symptom severity, this study improves interpretation of individual measurements in clinical and research contexts. Future work should validate reference intervals in diverse populations, integrate detailed symptom assessment, and evaluate whether modulation of GDF15 may hold therapeutic potential.

## Introduction

During pregnancy, the body undergoes profound and complex physiological and biochemical changes as it prepares and adapts to support the development of a growing foetus.^1^ Although natural, these changes often give rise to a wide range of undesired symptoms and conditions. Among the hormones associated with these pregnancy complications is Growth Differentiation Factor 15 (GDF15), which has gained attention for its dramatic surge during pregnancy and its role in nausea and vomiting.^2^, ^3^, ^4^

GDF15 is a cell regulatory protein expressed from most organs in response to cellular stress and disease, and it has been found to be elevated in varying degrees across a range of conditions, including various cancers, diabetes, obesity, organ damage, and ageing—but most notably during pregnancy.^5^ While GDF15 is elevated in many instances, its receptor, a heterodimer of *GDNF family receptor α-like* (GFRAL) and *rearranged during transfection* (RET) transmembrane receptor, is exclusively expressed in neurons of the area postrema and the nucleus tractus solitarius.^6^ These brain regions are known for detecting emetic signals from the blood and visceral sensory input, relaying them to neurons within the brain's vomiting centre.^7^ Dysfunction, e.g. as seen in area postrema syndrome, can cause persistent nausea, vomiting, and hiccoughs. Consistent with this neuronal localisation of GFRAL and RET, clinical trials with administration of a GDF15 analogue to humans induces nausea, vomiting and diarrhoea, with an association between increasing doses of the analogue and the type and severity of these adverse events.^8^ Conversely, research on Ponsegromab, a GDF15 antibody, demonstrated that through reducing GDF15 levels, the drug improved physical activity and appetite in patients with advanced cancer cachexia.^4^ Thus, GDF15 has a profound role in regulating nausea and vomiting through its neuronal receptors.

In alignment with the established link between nausea and GDF15, hyperemesis gravidarum (HG) has been associated with abnormally high GDF15 serum levels during pregnancy.^9^ While moderate nausea and vomiting is present in most pregnancies,^10^ HG represents an extreme form of this condition, and can result in significant weight loss, electrolyte imbalances, organ damage, and an increased risk of miscarriage.^11^ According to a meta-analysis,^12^ HG affects 1.1 percent (CI 95%: 0.8%–1.3%) of pregnancies during the first trimester. However, prevalence is subject to variations in diagnostic criteria across studies, as there is no consensus on the diagnostic criteria for HG. Likewise, there is no consensus on how to assess the severity of symptoms, however, the Pregnancy Unique Quantification of Emesis 24 (PUQE-24) score is widely used.^13^

While HG is a well-recognised clinical condition with a profound impact on pregnancy, we have only recently attained a better understanding of the aetiology through studies on GDF15. GDF15 has been identified as a key factor of HG using genetic approaches, with 4 independent variants near or within the GDF15 gene found to be the most highly associated single nucleotide polymorphisms (SNPs) in the maternal genome.^9^^,^^14^ Clinical studies have shown significantly elevated serum GDF15 levels in women with HG at the 12th week of gestation compared to normal pregnancies.^11^ However, by week 24, as symptoms have often resolved, GDF15 levels in HG cases were no longer significantly higher than those in normal pregnancies. More recent studies have shown that both fetoplacental unit production of GDF15 and maternal sensitivity to the hormone significantly contribute to the risk of HG.^3^ They also show evidence of maternal adaptation to high GDF15 levels and confirm, in a larger cohort, that elevated maternal GDF15 is associated with vomiting and HG during pregnancy.

While these advances have shed light on the role of GDF15 in relation to pregnancy and HG, significant challenges remain. One major issue is the substantial overlap in serum GDF15 levels between normal pregnancies and HG, making it difficult to assess the clinical relevance of GDF15 as a biomarker or therapeutic target.^3^ Additionally, the early pregnancy GDF15 trajectories are unknown as many studies are limited by single time-point sampling or wide gaps between samples, potentially missing important dynamics (e.g.^3^^,^^15^). Given the severe burden of HG, both in terms of its debilitating symptoms and its potential long term health consequences, it is paramount to uncover its underlying mechanisms.

This study aims to explore the role of GDF15 in early pregnancy by analysing longitudinal first trimester data from a large cohort of women with normal pregnancies, establishing a tentative reference interval, and assessing variations associated with adverse early pregnancy outcomes such as pregnancy loss and HG in another cohort.

## Methods

### Approvals and consent

The present study was approved by the Regional Committees on Health Research Ethics in the Capital Region of Denmark (H-21044817). We included data from two studies, the PEP Cohort^16^ and the VOMIT Study.^17^

All participants in the PEP Cohort were informed about the study both orally and in writing before inclusion and provided written informed consent in accordance with the Helsinki Declaration. The study was approved by the Regional Ethics Committee (H15018059) and the Danish Data Protection Agency (NOH-2015-042), and it was registered at ClinicalTrials.gov before the enrolment of the first participant (NCT02761772).

All participants in the VOMIT Study have signed informed consent, and the trial was registered at clinicaltrials.gov (NCT03785691). The trial was approved by the Regional Committees on Health Research Ethics in the Capital Region of Denmark (H-18047191), the Danish Medicines Agency (EudraCT 2018-002285-39), and the Danish Data Protection Agency (P-2019-75).

### Study cohorts: PEP and VOMIT

Data was obtained from two previous clinical trials: the PEP Cohort and the VOMIT Study. See Flowchart 1. A comparison between cohorts can be found in Supplementary Table S1.

### The PEP cohort

The Prospective Early Pregnancy (PEP) Cohort,^16^ included serum samples from pregnant women enrolled before day 55 of gestation and followed until approximately the end of the first trimester (before full 15 weeks’ gestation). In our study, serum samples from 194 women in the PEP Cohort were analysed for GDF15. Of these, 159 had naturally conceived, uncomplicated pregnancies that resulted in live births. Blood samples and PUQE-24 scores were collected at inclusion and every two weeks until pregnancy loss or completion of the first trimester occurred. All samples were stored at −80 °C until analysis. The earliest inclusion was at day 25 of gestation and latest entry at day 103. Every participant who completed the first trimester, had on average 3 or 4 visits for sampling.

Symptoms severity of nausea and vomiting was assessed every two weeks using the 24-h Pregnancy-Unique Quantification of Emesis (PUQE-24) questionnaire score, a widely used tool ranging from 3 to 15 that quantifies symptom severity over a 24-h period. Seven of these 159 women were excluded from analysis due to excessive PUQE-24 scores (≥13), indicative of HG, at their initial consultation (see Supplementary Figure S1 for analyses of these participants). As further workup, e.g. weight-loss registration or admission to the hospital was not conducted in this trial, the data could not be transferred to the VOMIT Study population. The remaining 152 pregnant women are referred to as the reference subcohort. Thirty-seven women had early pregnancy loss (EPL), divided as follows^18^: Missed Abortion (MA, n = 19), Anembryonic Pregnancy (AP, n = 9) Spontaneous Miscarriage (SA, n = 8), or Biochemical Pregnancy (BP, n = 1). These thirty-seven women are referred to as the EPL subcohort.

Inclusion happened between June 2016 and May 2017 at North Zealand Hospital, Denmark. A follow-up analysis of pregnancy and delivery outcomes was completed in 2018, identifying the participants who had an uncomplicated pregnancy and delivery.

### The VOMIT study

The VOMIT Study^17^ short for Validating the effect of Ondansetron and Mirtazapine In Treating hyperemesis gravidarum, was a randomised placebo-controlled double-blind multicenter trial conducted at seven hospitals in Denmark. The study enrolled women in gestational age 5–19 weeks with HG defined by PUQE-24 score ≥13 or PUQE-24 score ≥7 if accompanied by weight loss ≥5% or need for hospitalisation. Participants were randomly assigned 1:1:1 to treatment with placebo, mirtazapine or ondansetron. All participants in VOMIT were allowed rescue metoclopramide (max 10 mg x 3). In the present analysis, baseline serum samples from 53 pregnancies were available for GDF15 measurement. GDF15 levels were assessed at baseline and again after 14 days of treatment for the placebo group, however, only baseline samples were analysed in the mirtazapine and ondansetron groups, as treatment could affect the outcome. PUQE-24 scores were collected daily. All samples were stored at −80 °C until analysis. In addition to the original ethics committee approval, supplemental approval was obtained for the measurement of GDF15.

### PUQE-24

The PUQE-24 score ranges 3–15 and is based on questions quantifying nausea, vomiting, and retching in pregnant women.^19^

### Analysis of GDF15 by electrochemiluminescence immunoassay (ECLIA)

Due to the underreporting of specific GDF15 dimers by some of the most used ELISAs, the Roche Elecsys GDF15 electrochemiluminescence immunoassay (ECLIA. Calibrator: GDF-15 CS Elecsys, product number: 07125941190. Control: PreciControl Cardiac G2 Elecsys V4, product number: 04917049190. Reagent: GDF-15 Elecsys cobas e 100 V2, product number: 08946779190) was used to better capture all genotypes.^20^ The Roche (Roche Diagnostics, Basel, Switzerland) GDF15 ECLIA assay was performed as recommended by the manufacturer on a Roche COBAS e411 machine at Department of Clinical Biochemistry, Copenhagen University Hospital, North Zealand, Denmark. Samples were run in batch following calibration with approved controls prior and post the batch. Samples with concentrations above 20,000 pg/mL were set to automatic dilution ×5 and rerun for an exact result.

### Statistical analysis

The statistical approach is described in detail in the Statistical Supplement, and all analysis code is available (see Data sharing section).

Samples with missing GDF15 measurements or timestamps were excluded from analysis. When foetal sex was included as a covariate, observations with missing sex information were removed. No imputation was performed. Exploratory analyses were conducted to evaluate maternal age, maternal weight, parity, pre-pregnancy smoking status, pre-pregnancy alcohol consumption, prescription medication use, and chronic disease as covariates on the plateau parameter. Each covariate was evaluated in a separate model.

GDF15 concentrations were log-transformed to obtain normally distributed residuals and account for the wide dynamic range of values. Model selection was based on restricted maximum likelihood (REML) comparisons and semivariogram inspection. Data from the reference subcohort were analysed using a sigmoidal covariance pattern model with an exponential covariance structure to account for repeated measurements, as this provided the best fit compared to mixed models with random effects.

Nonlinear regression was applied to capture the sigmoidal trajectory of log-transformed GDF15 over time. The four-parameter model included plateau, basal level, inflection point in time and increase speed. Foetal sex effects were modelled as a dummy variable associated with plateau and inflection parameters.

Analyses were performed using the gnls function from the nlme package in R, with initial fitting using the nls function. Confidence intervals were derived by bootstrapping with the nlraa package. Mann–Whitney tests were used for comparisons of GDF15 levels between groups.

### Role of funders

Funders did not have any role in study design, data collection, data analyses, interpretation, or writing of report.

## Results

**Fig. 1:**
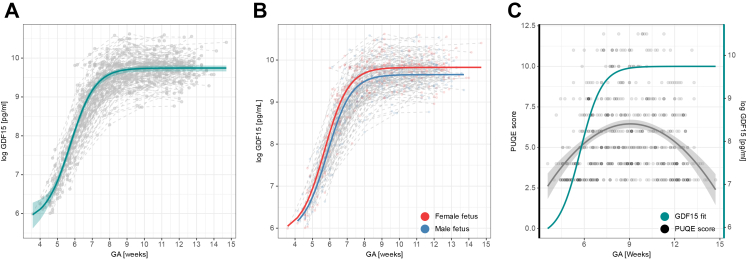
**Longitudinal GDF15 levels from the reference subcohort. (A)** GDF15 levels by gestational age in the reference pregnancy subcohort, fitted with a sigmoidal response and exponential covariance structure (green line, 95% CI shaded). Grey points represent individual measurements with connecting lines indicating repeated measures from the same woman. **(B)** Model fits stratified by foetal sex: female (red) and male (blue) **(C)** PUQE-24 scores (black, 95% CI shaded) overlaid with GDF15 trajectory from panel A (green).

The dynamics of GDF15 during early normal pregnancy are depicted in Fig. 1A, with a significant sigmoidal model fit (P < 0.01). Fig. 1A shows a surge in GDF15 levels between study initiation and week 7, representing the most pronounced increase during the study period. Following this initial surge, the curve flattened between weeks 7–8, with GDF15 stabilising at a plateau by week 9.

Stratification by foetal sex (Fig. 1B) revealed higher GDF15 levels in pregnancies with female foetuses. At later stages, GDF15 concentrations were approximately 19% higher in pregnancies with female foetuses (P < 0.01). In addition, the rise in GDF15 occurred approximately one day later in pregnancies with male foetuses (P < 0.02). Exploratory analyses evaluated the influence of maternal characteristics on GDF15 trajectories. Maternal age showed no measurable association with GDF15 levels. Maternal weight was negatively associated with the plateau parameter (estimate = −0.01, P < 0.001), corresponding to an approximate 1% lower GDF15 concentration per kilogramme of maternal weight. Additional analyses incorporating parity, pre-pregnancy smoking status (yes/no), pre-pregnancy alcohol consumption (yes/no), prescription medication use (yes/no), and chronic disease (yes/no) as covariates on the plateau parameter showed statistically significant associations for multiparity (estimate = −0.145, P = 0.017) and pre-pregnancy alcohol consumption (estimate = 0.150, P = 0.004). Pre-pregnancy smoking status (estimate = 0.034, P = 0.28), medication use (estimate = 0.155, P = 0.096), and chronic disease (estimate = −0.015, P = 0.394) were not significantly associated with the plateau parameter. The magnitude of these effects was small relative to the overall gestational increase in GDF15 and did not materially alter the estimated trajectories or overall conclusions.

PUQE-24 scores from the reference subcohort, in combination with the GDF15 fit from Fig. 1A, are presented in Fig. 1C. Symptom severity, as measured by PUQE-24 scores, followed a concave trajectory, initially increasing from the time of inclusion, peaking around week 8, and gradually declining thereafter.

Symptom trajectories broadly followed the rise and plateau of GDF15 levels, with a peak in symptom severity aligning with the plateau phase of GDF15 levels. Notably, symptom resolution occurred as GDF15 levels stabilised. When stratified by foetal sex, there was no significant difference between PUQE-24 scores (Supplementary Figure S2).

Raw GDF15 data are presented from the PEP Cohort in the form of weekly medians with 95% confidence intervals (tentative reference intervals), combined and stratified by foetal sex (Table 1), illustrating the physiological rise in GDF15 during early normal pregnancy, from median levels of 602 pg/mL (female foetuses) and 635 pg/mL (male foetuses) at gestational week 4 to a plateau around week 9 for female foetuses (∼19,000 pg/mL) and week 10 for male foetuses (∼17,000 pg/mL).

**Table 1:** Serum GDF15 values.

Week	Female			Male			All		
	N measurements	Median [pg/ml]	95% confidence interval	N measurements	Median [pg/ml]	95% confidence interval	N measurements	Median [pg/ml]	95% confidence interval
4	16	602	[523; 930]	18	635	[546; 769]	34	630	[552; 714]
5	28	1570	[1125; 2172]	24	1591	[1055; 1783]	52	1591	[1259; 1789]
6	41	8205	[6045; 9399]	39	5016	[3882; 5678]	80	5861	[5066; 7290]
7	40	13,409	[11,989; 15,944]	36	10,712	[8530; 12,595]	76	12,294	[10,617; 13,593]
8	37	18,015	[13,323; 18,506]	36	14,471	[12,663; 15,503]	73	15,199	[13,323; 17,794]
9	34	19,251	[16,319; 20,443]	39	13,910	[12,199; 16,500]	73	16,159	[13,910; 17,716]
10	32	19,323	[17,270; 19,932]	28	16,790	[15,431; 18,131]	60	18,098	[16,448; 19,371]
11	34	18,794	[17,153; 19,925]	30	16,372	[14,269; 18,498]	64	17,630	[16,304; 19,245]
12	33	17,449	[15,474; 19,268]	35	17,713	[15,548; 18,736]	68	17,600	[16,786; 18,617]
13	6	19,208	[14,252; 31,179]	9	16,097	[13,306; 21,666]	15	16,097	[14,404; 21,666]
14	3	26,465	[14,420; 33,019]	NA	NA	NA	NA	NA	NA

Raw GDF15 data are presented from the PEP Cohort in the form of weekly medians with 95% confidence intervals (tentative reference intervals), combined and stratified by foetal sex.

NA, not applicable.

**Fig. 2:**
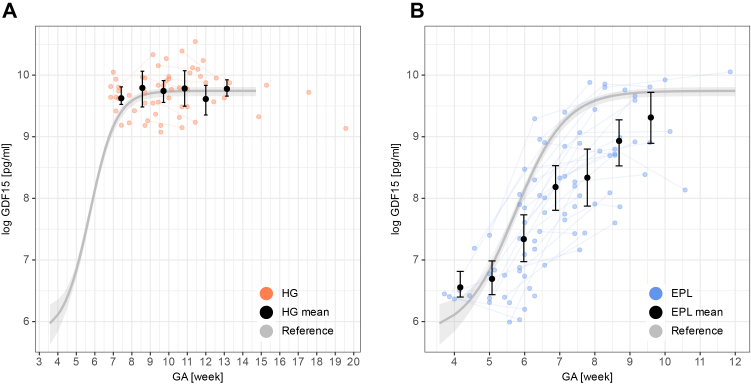
**Divergent trajectories of GDF15 in hyperemesis gravidarum (HG) and early pregnancy loss (EPL).** GDF15 levels in HG (VOMIT Study, panel A) and EPL (the EPL subcohort, panel B) compared with the reference subcohort (grey, Fig. 1A). Coloured points indicate individual measurements, with connecting lines representing repeated measures from the same participant. Black dots show interval means with 95% CI.

The reference subcohort (Fig. 1A) was compared to the EPL subcohort and women with HG from the VOMIT Study (Fig. 2A and B).

**Fig. 3:**
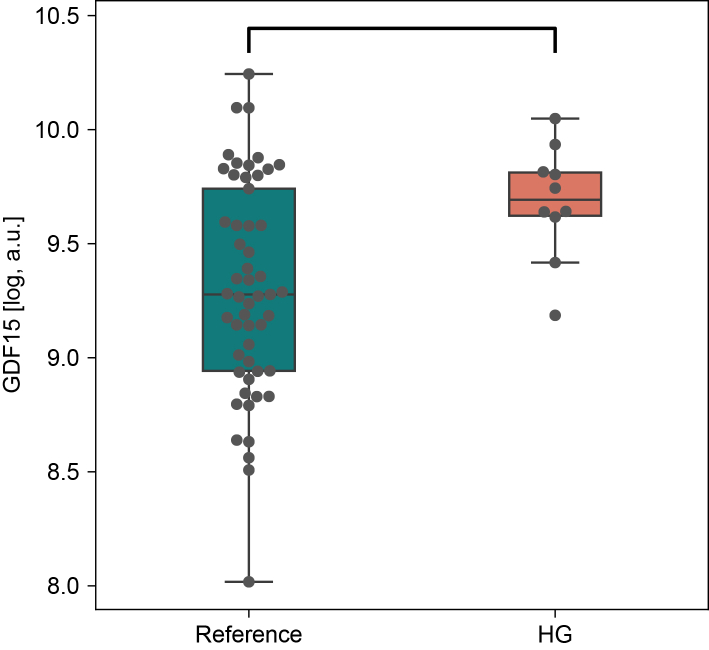
**Elevated GDF15 in Hyperemesis Gravidarum (HG) compared to controls.** Mann–Whitney test of the early period (day 48–52) with a significant difference (P = 0.013) in GDF15 levels between the pregnant reference subcohort (green) and HG (orange). The figure also illustrates the limited number of patients with HG in the early stages of the VOMIT Study as well as the substantial overlap of values.

In women with HG, GDF15 levels were consistent with an early plateau by the time of inclusion, as shown by individual measurements and interval medians in Fig. 2A. By gestational week 8, the reference subcohort and HG began to overlap, indicating no significant differences between the two groups at this stage. An analysis of the early period (day 48–52) by the Mann–Whitney test revealed a significant difference (P < 0.02) in GDF15 levels between HG and the reference subcohort (Fig. 3), but not with Welch's t-test (P = 0.187).

Beyond the fitted reference subcohort trajectory, HG values remained indicative of a maintained plateau. No significant effect of treatment with either mirtazapine (P = 0.70) or ondansetron (P = 0.83) was observed (Supplementary Figure S3).

Women in the EPL subcohort exhibited lower GDF15 levels and a slower rise compared with the reference subcohort (Fig. 2B). Confidence intervals of the estimated means did not overlap with those of the reference subcohort from shortly after week 5 to approximately week 9. The curve flattened and plateaued at approximately the same time as in the reference subcohort. A visual representation of EPL stratified by classification is provided in Supplementary Figure S4. GDF15 levels in some cases of missed abortion, representing the majority of EPL cases, were comparable to those observed in normal pregnancies. Overall variability and heterogeneity across EPL types was high.

## Discussion

By analysing longitudinal data from two large cohorts of women in early pregnancy, we gained insights into the temporal dynamics of GDF15 and further extended our analyses to encompass HG and EPL. Using repeated measurements rather than cross-sectional sampling, we identified a sigmoidal pattern characterised by a rapid early rise followed by stabilisation, refining prior descriptions of GDF15 trajectories in early pregnancy.

Fejzo et al. previously demonstrated the relationship between gestational age and increasing GDF15 levels in the first trimester using a linear regression model based on single timepoint samples from women with and without HG.^3^ In contrast, by applying a four-parameter sigmoidal model, our data revealed an initial rapid increase in GDF15 levels, followed by stabilisation and plateau (Fig. 1A). Although exploratory analyses identified statistically significant associations between the plateau parameter and maternal weight, multiparity, and pre-pregnancy alcohol consumption, the magnitude of these effects was small relative to the overall gestational increase in GDF15 and did not substantially alter the estimated trajectories or overall conclusions (see Statistical Supplement). Notably, in the absence of direct baseline data, the model predicted a GDF15 concentration of 319 pg/mL, approximately half of the mean level reported by Welsh et al. (628 pg/mL, 95% CI 616–641) in a large cohort of healthy non-pregnant women under 30 years of age.^21^ However, due to the lack of data in the earliest weeks of gestation, the model's precision cannot be used to make reliable predictions at this stage. Despite substantial inter-individual variability, the overall sigmoidal pattern of GDF15 elevation was preserved, refining our understanding of GDF15 fluctuations. These insights highlight a critical window for optimal sampling and the necessity of longitudinal sampling in future research. The reference subcohort further provides a starting point to evaluate reference intervals for early pregnancy GDF15 levels, though future work should validate reference intervals in diverse populations and larger sample sizes with more timepoints.

Our finding of GDF15 plateauing does not imply stability throughout gestation. A small longitudinal study^11^ observed rising GDF15 levels from approximately 10 to 12 ng/mL between the first and second trimester, while a larger cross-sectional study^22^ reported substantially higher concentrations, approaching 50 ng/mL, in late pregnancy, concluding a linear increase based on multiple regression analyses. Marjono et al.^23^ similarly reported increasing GDF15 levels later in pregnancy using cross-sectional sampling, showing a second peak around week 27 of gestation. Likewise, Andersson-Hall et al.,^24^ who followed women longitudinally across all three trimesters, also described a second peak between the second and third trimesters, supporting a rise beyond mid-gestation. In our dataset, four women had late entries into week 20, preceding the second peak reported in these studies. These samples aligned with the plateau fit for HG but showed substantial variance and were underpowered for any definitive conclusion. Taken together, existing evidence suggest that GDF15 continues to rise later in pregnancy. However, the timing, magnitude and shape of this increase requires detailed longitudinal studies, as individual variability is considerable and single timepoint sampling overlooks important dynamics, as we have demonstrated to be the case in early pregnancy.

Examination of clinical relevance showed that pregnancy symptoms quantified by PUQE-24 in the reference subcohort worsened from inclusion, peaking around eight weeks’ gestation, and gradually resolved thereafter, roughly corresponding to the rise and plateau in GDF15 levels. Notably, symptom resolution coincided with the stabilisation of GDF15, potentially reflecting a rapid desensitisation to constantly elevated levels, consistent with a mechanism described by Fejzo et al.^3^ These findings corroborate a prospective study from 1993^10^ that had 363 women record nausea and vomiting onset as early as 3 weeks post-last menstrual period, with symptoms peaking by week 8 and resolving by week 12 in most cases. Consistent with earlier reports,^24^ we confirmed higher GDF15 levels in pregnancies with female foetuses throughout the first trimester. However, we did not observe a significant relation between the change in GDF15 and PUQE-24 score when stratified by foetal sex. These findings provide insights into symptom progression and resolution that may inform treatment planning, study design, and patient consultations.

In the EPL subcohort, we observed a trajectory characterised by lower GDF15 levels and a slower rise compared with the reference subcohort, with significant differences between weeks 5 and 9 of gestation. Beyond this point, differences were no longer statistically significant, and confidence intervals widened due to fewer available measurements. GDF15 levels are strongly associated with gestational duration, impaired fetoplacental function is therefore likely linked to both lower GDF15 concentrations and earlier pregnancy demise, resulting in an underrepresentation of low values at later gestational ages. As the fetoplacental unit is the primary source of circulating GDF15,^3^ a reduction or cessation of its function could explain the flattened or declining trajectories seen before miscarriage. In keeping with this, GDF15 levels in some cases of missed abortion remained within the normal range up until pregnancy loss, whereas in others the typical early pregnancy rise was absent. Overall variability across EPL types was high. Although GDF15 levels were consistently lower in the EPL group, these observations reflect association and cannot establish causality.

In women with HG from the VOMIT study, GDF15 trajectories exhibited a distinct early plateau by the time of inclusion compared with normal pregnancies, suggesting an earlier hormonal rise that was short-lived and disappeared by 7 weeks' gestation. This finding is based on a limited number of measurements and was supported by non-parametric analysis (Mann–Whitney test), but not by Welch's t-test, showing that, although GDF15 values in patients with HG ranked significantly higher than those in the reference subcohort, the variance in the reference subcohort was too large to yield a significant t-statistic. This likely reflects sampling around the inflection point of the reference GDF15 trajectory, where between-subject variance is high. Six women from the HG group showed slightly decreasing GDF15 levels between first and second visit, which could speculatively be explained by the permitted use of metoclopramide. However, both increases and decreases were observed among the reference subcohort, indicating that individual short-term trajectories are not strictly monotonic, even though the overall population-level trajectory tends to increase before plateauing. We observed no significant change in GDF15 levels with mirtazapine or ondansetron treatment. The overall timing of inclusion in the VOMIT study aligns with previous reports that HG-related hospitalisations typically begin around week 5 and peak at week 8,^25^ supporting the hypothesis that the initial GDF15 surge occurs just before clinical presentation.

Several limitations should be considered when interpreting these findings. First, sampling in the earliest weeks of gestation was limited across both cohorts, restricting direct observations of baseline GDF15 concentrations and the earliest dynamics. Especially in the HG group, with no sampling prior to week 6 of gestation. This is an inherent consequence of the inclusion criteria for the VOMIT study where participants were enrolled as late as gestational week 19. Typically, participants were enrolled after they had tried coping with symptoms before being referred to hospital. Secondly, missing data in the HG cohort was not entirely random, since most drop-outs were due to treatment failure. This missingness may bias observed trajectories toward earlier stabilisation and underrepresenting prolonged disease course. Third, although all samples analysed were serum and stored frozen under identical conditions prior to analysis, some inter-cohort heterogeneity in sample handling and sample age is unavoidable and may contribute to variability, though such effects would be expected to be nondifferential. Finally, the representativeness of the included cohorts is inherently difficult to fully ascertain. A strength of the study is that participants in both cohorts were recruited from the same geographical region and healthcare system, within a relatively homogeneous population predominantly consisting of Caucasian women, which supports internal comparability. While a formal demographic comparison between cohorts was limited, previous work on the reference cohort has demonstrated that pregnancy cohorts drawn from this setting show good agreement with the Danish National Birth Cohort with respect to key baseline characteristics.^16^ This suggests that the underlying populations are broadly representative. A comparrison of cohorts can be found in Supplementary Table S1. Nonetheless, we acknowledge that some selection bias cannot be excluded, particularly in the HG cohort where inclusion required hospital referral.

In conclusion, this study reports detailed early pregnancy trajectories of GDF15 using a longitudinal design, demonstrating a sigmoidal rise and plateau, with distinct patterns in HG and EPL. These findings refine current understanding of GDF15 dynamics in pregnancy and define optimal sampling windows and tentative reference intervals in uncomplicated early pregnancy. Further research is warranted to clarify the timing and clinical significance of GDF15 changes, particularly in the earliest stages of HG, and to extend longitudinal sampling into later gestation. Such work may help inform future diagnostic and therapeutic strategies.

## Contributors

JSS, AO, JFP, EL, and MPG contributed to the conception and design of the work. GDF15 analysis by TH. JFP, AO, JSS and MSS accessed and verified the underlying data. Statistical analysis by JSS and MSS. Writing original draft by JSS. All authors assisted in writing, reviewing and editing as well as interpreting data. Funding by EL, MPG and TJG. All authors have agreed to be accountable for all aspects of the work.

All authors read and approved the final version of the manuscript.

## Data sharing statement

Anonymised participant data can be made available on request to the corresponding author with a mutually agreed data sharing agreement.

Code used for the analyses presented in this manuscript is available at www.GitHub.com: https://github.com/mssven/longitudinal-gdf15.

## Declaration of generative AI and AI-assisted technologies in the manuscript preparation process

During the preparation of the initial draft of this work, the author JSS used Microsoft Copilot for minor assistance with language phrasing and improvement of text clarity. After using this tool, all authors reviewed and edited the content as needed and take full responsibility for the content of the published article.

## Declaration of interests

EL had travel expenses from Gedeon Richter, Merck, and Astelas and honoraria for lectures from Novo Nordisk and Gedeon Richter.

JSS, AO, JFP, MSS, TH, TJG, MPG: nothing to declare.

## Appendix A Supplementary data

Statistical supplement

Supplementary Figure S1 **GDF15 levels in excluded participants.** Seven of the 159 women were excluded from the analysis due to elevated PUQE‑24 scores (≥13), indicative of hyperemesis gravidarum, at the initial consultation.

Supplementary Figure S2 **PUQE-24 scores stratified by fetal sex.** PUQE-24 scores (dots) with mean value stratified by fetal sex: female (red) and male (blue) with 95% CI (grey, shaded).

Supplementary Figure S3 **Effect of treatment with mirtazapine or ondansetron on GDF15 levels in HG.** No significant changes over time or between groups. Paired first and second visits are shown (lines connect individuals, unbalanced sampling not accounted for). Linear mixed modelling showed no effect of mirtazapine (P = 0.70) or ondansetron (P = 0.83).

Supplementary Figure S4 **GDF15 levels differ with type of Early Pregnancy Loss (EPL).** GDF15 levels in the EPL subcohort. Points represent individual measurements with connecting lines indicating repeated measures from the same woman. Anembryonic pregnancy (AP, orange), Missed Abortion (MA, blue), Spontaneous Abortion (SA, purple), Biochemical Abortion (BP, green). Reference subcohort from figure 1A for comparison (grey, shaded 95% CI).

Supplementary Table S1 **Cohort comparison at the time of inclusion compared with the 2017 nationwide Danish birth data.** P-values for comparisons between cohorts were calculated using Welch’s t-test for continuous variables and chi-square or Fisher’s exact tests for categorical variables. ∗Weight in VOMIT is at inclusion, during pregnancy.

Flowchart (A) Participant inclusion from the Prospective Early Pregnancy (PEP) Cohort, divided in this study into the pregnant reference subcohort and the Early Pregnancy Loss (EPL) subcohort. (B) Participant inclusion in the Validating the Effect of Ondansetron and Mirtazapine in Treating Hyperemesis Gravidarum (VOMIT) Study. n = number of participants, N = number of measurements.
