# Supplementary material for: Early pregnancy GDF15 trajectories and their association with normal gestation, pregnancy loss, and hyperemesis gravidarum
Source: eBioMedicine. 2026 Aug 6;131:106428. doi: 10.1016/j.ebiom.2026.106428 (PMC13475652; doi:10.1016/j.ebiom.2026.106428)
Supplement: Supplementary file 2 [file mmc3.pdf]

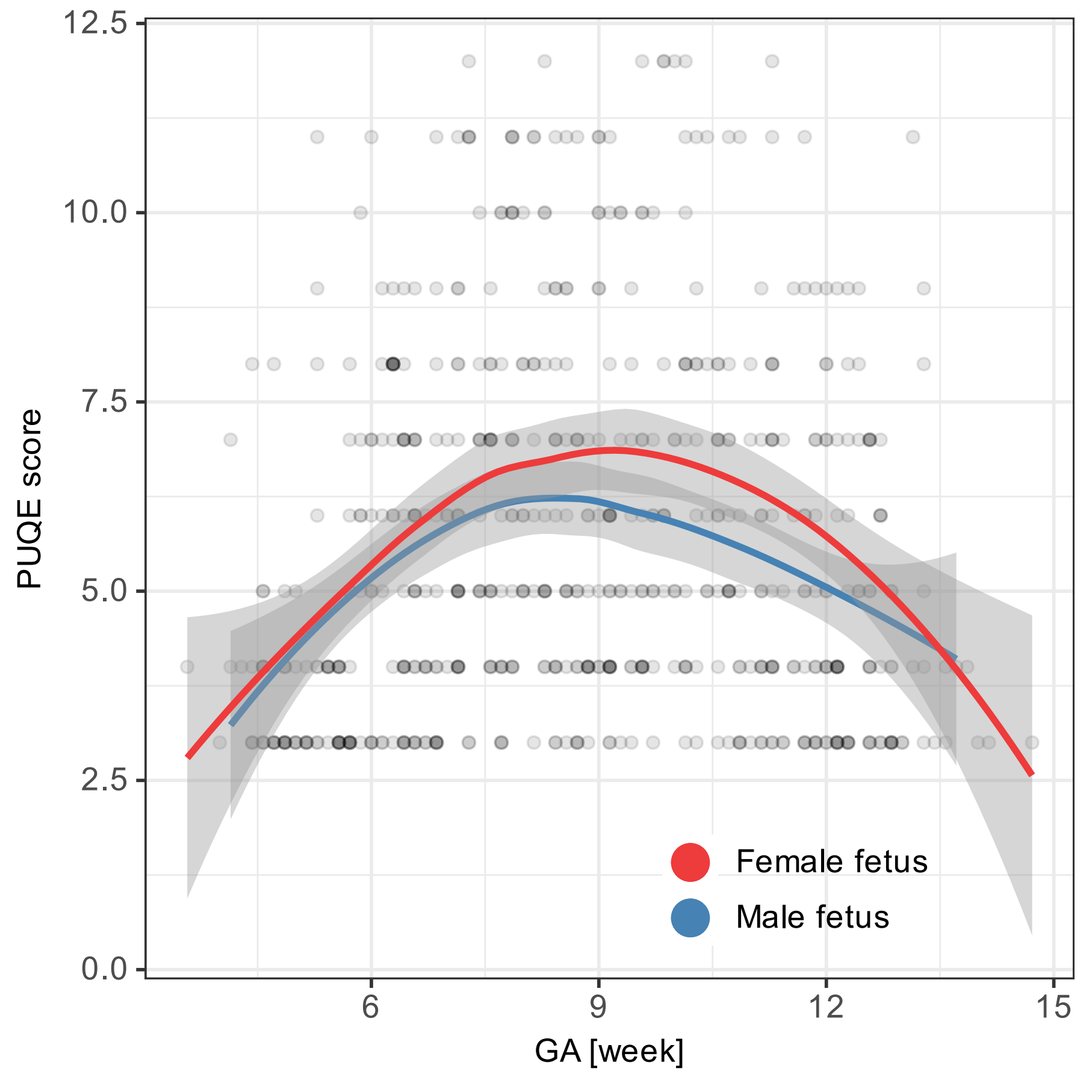

12.5
10.0
7.5
5.0
2.5
0.0
PUQE score
6
9
12
15
GA [week]
Female fetus
Male fetus
