# Supplementary material for: Early pregnancy GDF15 trajectories and their association with normal gestation, pregnancy loss, and hyperemesis gravidarum
Source: eBioMedicine. 2026 Aug 6;131:106428. doi: 10.1016/j.ebiom.2026.106428 (PMC13475652; doi:10.1016/j.ebiom.2026.106428)
Supplement: Supplementary file 3 [file mmc4.pdf]

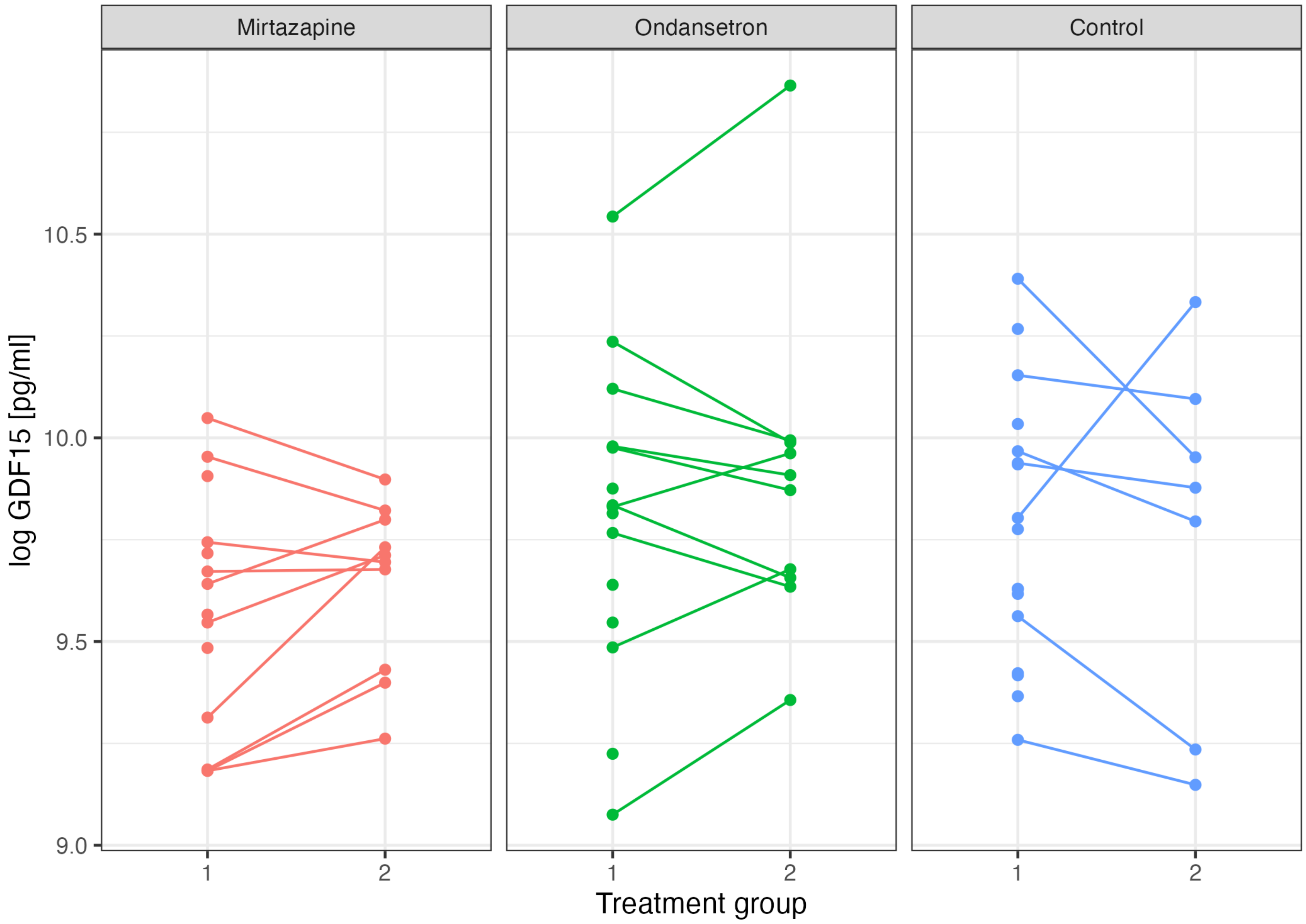

Mirtazapine
Ondansetron
Control
log GDF15 [pg/ml]
10.5
10.0
9.5
9.0
1
2
Treatment group
