# Supplementary material for: Early pregnancy GDF15 trajectories and their association with normal gestation, pregnancy loss, and hyperemesis gravidarum
Source: eBioMedicine. 2026 Aug 6;131:106428. doi: 10.1016/j.ebiom.2026.106428 (PMC13475652; doi:10.1016/j.ebiom.2026.106428)
Supplement: Supplementary file 4 [file mmc5.pdf]

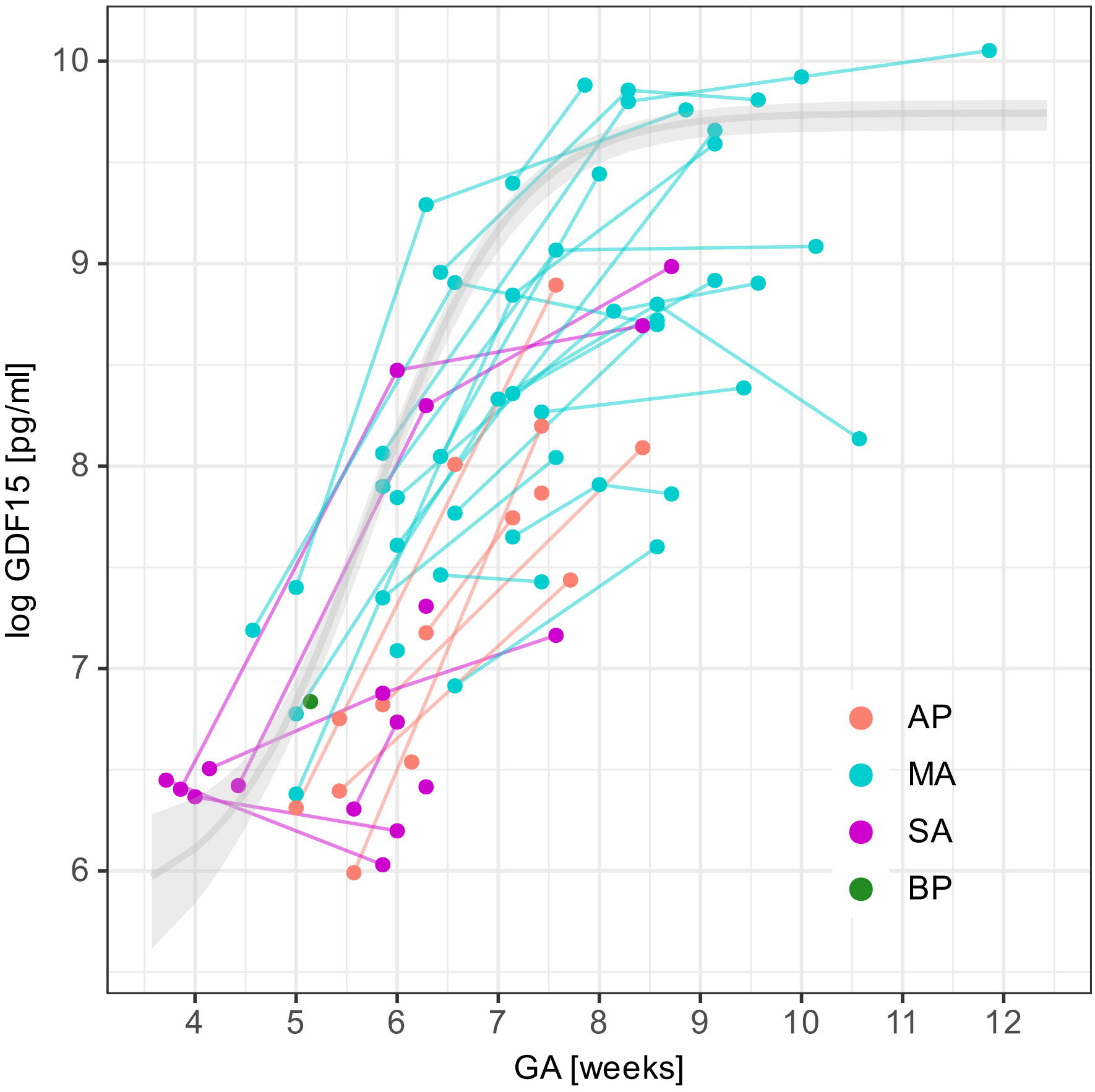

log GDF15 [pg/ml]
10
9
8
7
6
GA [weeks]
4
5
6
7
8
9
10
11
12
AP
MA
SA
BP
