# Supplementary material for: Early pregnancy GDF15 trajectories and their association with normal gestation, pregnancy loss, and hyperemesis gravidarum
Source: eBioMedicine. 2026 Aug 6;131:106428. doi: 10.1016/j.ebiom.2026.106428 (PMC13475652; doi:10.1016/j.ebiom.2026.106428)
Supplement: Supplementary file 5 [file mmc7.pdf]

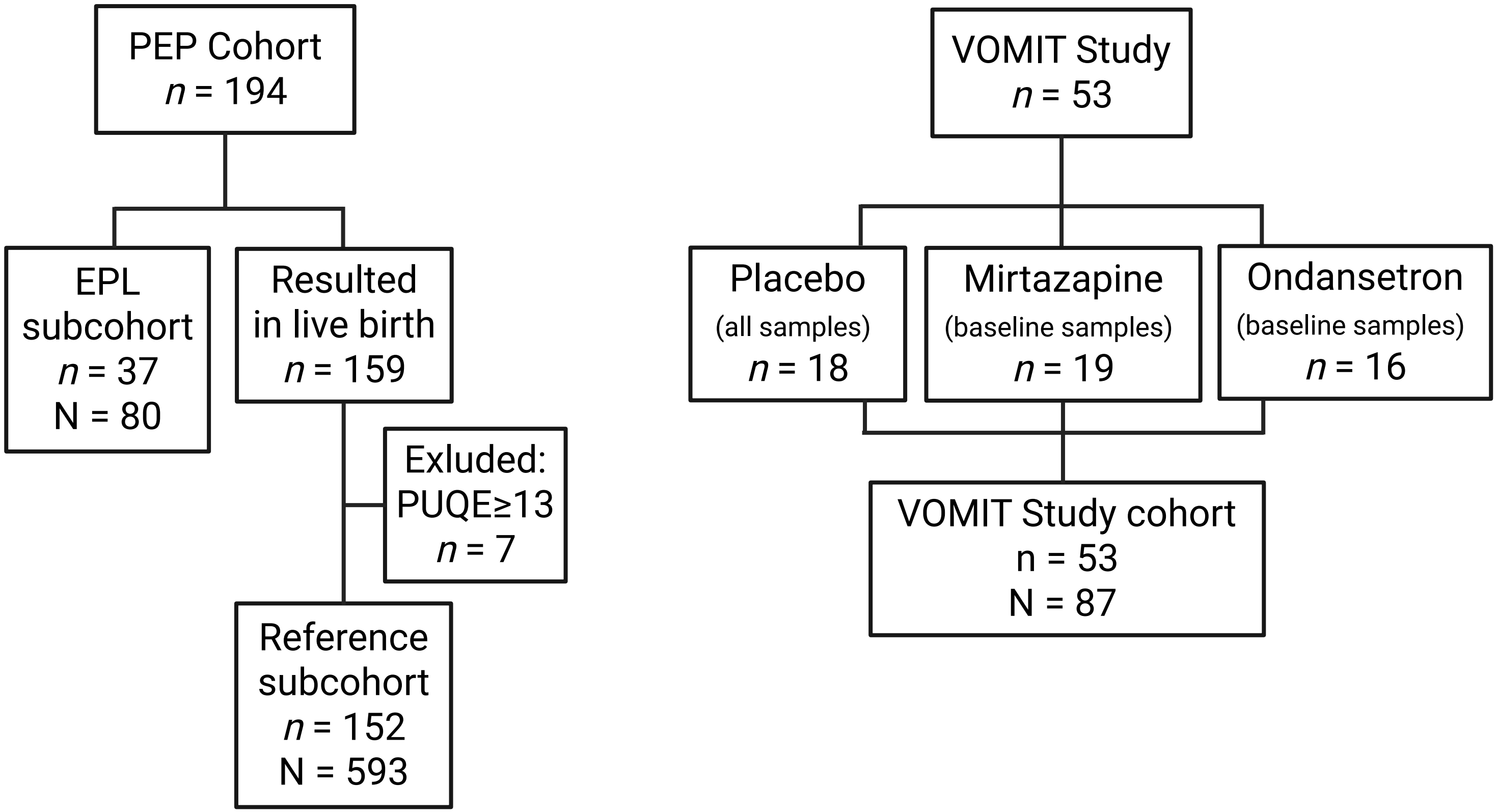
PEP Cohort
n = 194
EPL subcohort
n = 37
N = 80
Resulted in live birth
n = 159
Exluded:
PUQE≥13
n = 7
Reference subcohort
n = 152
N = 593
VOMIT Study
n = 53
Placebo
(all samples)
n = 18
Mirtazapine
(baseline samples)
n = 19
Ondansetron
(baseline samples)
n = 16
VOMIT Study cohort
n = 53
N = 87
