# Supplementary material for: Early pregnancy GDF15 trajectories and their association with normal gestation, pregnancy loss, and hyperemesis gravidarum
Source: eBioMedicine. 2026 Aug 6;131:106428. doi: 10.1016/j.ebiom.2026.106428 (PMC13475652; doi:10.1016/j.ebiom.2026.106428)
Supplement: Supplementary file 6 [file mmc1.docx]

## Statistical supplement

The raw GDF15 measurements were filtered for missing values prior to the regression analysis. Samples with missing values in either GDF15 measurements or missing timestamps were removed from the analysis. Samples with missing values in the fetal sex variables were removed if sex appears as a covariate in the statistical model. No imputations were performed. The GDF15 measurements were log-transformed to obtain normally distributed residuals in the statistical analysis. Normality of the residuals was assessed using both a Q-Q plot and a histogram of Cholesky transformed residuals, obtained with the nlme::residuals(gnls_fit, method=”normalized”), thereby accounting for within-subject correlation.

Additionally, the dynamics of GDF15 plasma concentration span orders of magnitude, which makes it adequate to model the response in log space.

The study is based on repeated plasma measurements from the same individuals, which then requires a repeated measurements statistical approach. Additionally, the measurements are not obtained at fixed timepoints, meaning that the experimental design contains unbalanced time. Linear mixed models with explicitly modelled covariance pattern were employed to analyze the data, yielding the possibility of explicitly modelling the correlation structure of residuals.

Covariance‑pattern models were used to analyze the data, yielding the possibility of explicitly

modelling the correlation structure of residuals. Models with equal fixed effects were compared using restricted maximum likelihood estimate (REML). Covariance pattern models provided a superior fit relative to mixed‑effects models, as indicated by semivariograms and higher REML values. An exponential covariance structure offered the best fit, outperforming mixed-effects model with either random intercepts or a more complex group of

random effects.

The response shape of log GDF15 over time was inspected using a smoothed mean function. This yielded a sigmoidal shape. The adequate statistical model thus needed to capture the repeated measurements and a nonlinear response, while adequately fitting the covariance structure between residuals within the same individual. The statistical analysis was performed using the gnls function from the nlme package in R. All functions were fitted first with the nls function. This fit provided initial guesses for parameter values for the gnls function. Since the p-values estimated by native functions of nlme are ill-estimated, with hypothesis tests for this class of models being approximative, we employed additional bootstrapping to inspect significance of estimated model parameters. This was done using the package nlraa. Functions from the nlraa package was also used to estimate confidence bands around the fitted functions. The following is a description of the analysis specific to each figure.

Figure 1A: The dynamics of GDF15 during early normal pregnancy is depicted in Figure 1A. It shows a sigmoidal four parameter non-linear regression model describing log transformed GDF15 as a function of time with the mathematical expression. The four parameters account for the plateau that GDF15 presumably reaches ($b_{plateau}),$the initial basal level ($b_{basal})$, the inflection point in time where GDF15 increases dramatically ($b_{inflection}$) and increased speed of GDF15 ($b_{increase}).$

$$y_{\log gdf15}(t)=\frac{b_{plateau}-b_{basal\_level}}{1+exp(-b_{increase}\left( t+b_{inflection} \right))}+b_{basal\_level}$$

Parameters-results of the mathematical expression being: plateau = 9·744, inflection = -40·1, timescale increase = 0·199, estimate for basal level = 5·784. The covariance of residuals is adequately fitted by an exponential structure, accounting for repeated measurements of the same participant over time.
95% confidence interval is estimated using bootstrapping and refitting.

Figure 1B: Same functional form as analysis in Figure 1A, but with two parameters associated with fetus sex differences. Sex is included as a dummy variable associated with two parameters, the first is a change in the plateau and the second is a change in the inflection time point. The p-values obtained from the analytical t-test is lower than the one estimated by bootstrapping. The functional form was:

$$y_{\log gdf15}(t)=\frac{b_{plateau}-b_{basal}+b_{plateau:female}D_{female}}{1+exp(-b_{increase}\left( t+b_{inflection}+ b_{inflection:female}D_{female} \right))}+b_{basal\_level}$$

The parameter $b_{plateau:female}$= 0·172 corresponding to a female fetus giving 19 percent higher

blood plasma GDF15 at later stages in the pregnancy (P=0·002), and the parameter $b_{inflection:female}$=1.04 (P=0·014) corresponds to the rise in GDF15 being delayed by one day when the fetus is male.

Figure 1C: PUQE-24 scores from the 152 women in the reference cohort from the PEP Cohort with normal pregnancies, overlaid with the GDF15 fit from Figure 1A.

Figure 2: Fit to reference subcohort compared with time-dependent mean of both the VOMIT cohort (A) and the EPL cohort (B). Mean values are calculated for sub-intervals of time. Error bars depict 95% confidence intervals of the means estimated with bootstrapping. (A) VOMIT cohort with 50 women showing an approximately constant mean. There are no measurements with low early values, due to later enrollment. (B) EPL cohort with 37 women showing a sigmoidal increase in GDF15, but lower levels compared to the reference subcohort.

Figure 3: Mann-Whitney between the reference subcohort and VOMIT Study samples obtained between day 48 and day 52. During this period, 53 samples from the reference subcohort were included in contrast to 10 samples from women with HG. There were no repeated measurements within this time interval. Note, we repeated the analysis using an unpaired t-test with unequal variances (Welch’s t-test).

Supplementary Figure 1. Seven of the 159 women were excluded from the analysis due to elevated PUQE‑24 scores (≥13), indicative of hyperemesis gravidarum, at the initial consultation. These are plottet against normal pregnancies. No further analysis.

Supplementary figure 2: PUQE-24 scores stratified by fetal sex. PUQE-24 scores from the 152 women in the reference cohort from the PEP Cohort with normal pregnancies, stratified by fetal sex.

Supplementary Figure 3. Effect of treatment with mirtazapine or ondansetron on GDF15 levels in HG.
No significant changes in GDF15 levels were observed over time in any treatment group, and no significant differences in change were detected between groups. The figure ignores the unbalanced sampling times (at plateau phase) and shows the first and second visit, with lines indicating paired measurements within the same subject. The effect of intervention was tested using a linear mixed model with an estimated control intercept and additional parameters associated with either treatment, showing no significant effect of either mirtazapine (p = 0.7) nor ondansetron (p = 0.83).

Supplementary figure 4. Plot of EPL with reference subcohort for comparison, without further statistical analysis.

Exploratory analysis of covariates:
We performed exploratory analyses to assess the potential influence of maternal characteristics and other covariates on model residuals and parameter estimates. Maternal age showed no measurable effect on GDF15 trajectories. BMI demonstrated a weak negative association with GDF15 levels. However, further investigation revealed that this effect was primarily driven by maternal weight rather than height. When BMI was decomposed into weight and height, height showed no apparent association, whereas weight exhibited a modest negative effect that was most pronounced at later gestational ages. Consequently, weight was included as a covariate on the plateau parameter. The estimated effect was −0.01 (p < 0.001), corresponding to an approximate 1% decrease in GDF15 per kilogram of maternal weight. Nevertheless, substantial inter-individual variability remained.

We further explored the effects of parity, smoking status, alcohol use, medication use, and chronic disease by including each covariate as an offset on the final plateau parameter in separate models, with fetal sex included as an adjustment factor in all analyses. Alcohol consumption prior to pregnancy (estimate = 0.150, p = 0.0036) and multiparity (estimate = −0.145, p = 0.017) were associated with statistically significant differences in the final plateau level. Smoking (estimate = 0.034, p = 0.28), medication use (estimate = 0.155, p = 0.096), and chronic disease (estimate = −0.015, p = 0.394) were not significantly associated with the plateau parameter. Importantly, the magnitude of these effects was small compared with the substantial increase in GDF15 observed across gestation, with a limited influence on the estimated trajectories and did not materially alter the overall findings or conclusions. Given the substantial inter-individual variability and the minimal impact of covariate adjustment, we chose not to include these variables in the primary model. This approach was taken to maintain model parsimony and interpretability while preserving the robustness of the main results.
Note: Alcohol refers to any alcohol consumption prior to pregnancy. Smoking refers to any smoking habit prior to pregnancy. Parity is a binary variable indicating whether or not this was the first pregnancy. Medication refers to any prescription medication use. Chronic disease refers to the presence of any noted chronic disease.
