# Supplementary material for: Early pregnancy GDF15 trajectories and their association with normal gestation, pregnancy loss, and hyperemesis gravidarum
Source: eBioMedicine. 2026 Aug 6;131:106428. doi: 10.1016/j.ebiom.2026.106428 (PMC13475652; doi:10.1016/j.ebiom.2026.106428)
Supplement: Supplementary file 7 [file mmc6.docx]

**Supplementary table 1**

|  | **PEP Cohort** | **VOMIT cohort (full cohort)** | **Danish 2017 births** | **PEP VS VOMIT** | **PEP VS DANISH 2017 BIRTHS** | **VOMIT VS DANISH 2017 BIRTHS** |
| --- | --- | --- | --- | --- | --- | --- |
| n | 122 | 59 | 60871 | P-value | P-value | P-value |
| Age, years mean (SD) | 29.3 (4.1) | 29.1 (4.8) | 30.3 (5.0) | 0.82 | <0.001 | <0.001 |
| <30 years (%) | 76 (62.3) | 34 (57.6 | 27694 (45.5) | 0.56 | <0.001 | <0.001 |
| 30–35 years (%) | 33 (27.0) | 19 (32.2) | 23547 (38.7) |  |  |  |
| >35 years (%) | 13 (10.7) | 6 (10.2) | 9630 (15.8) |  |  |  |
| BMI, kg/m2 mean (SD)* | 24.1 (4.4) | 25.0 (5.0)* | 24.8 (8.2) | 0.16* | 0.42 | 0.88* |
| Normal weight (%) | 76 (62.3) | 31 (53.4)* | 35189 (57.8) | 0.08 | <0.05 | 0.57 |
| Underweight (%) | 3 (2.5) | 2 (3.4)* | 2579 (4.2) |  |  |  |
| Pre-obese (%) | 34 (27.9) | 15 (25.9)* | 12683 (20.8) |  |  |  |
| Obese (%) | 9 (7.4) | 10 (17.2)* | 8009 (13.2) |  |  |  |
| Missing | NA | 1 | 2411 (4.0) |  |  |  |
| Former pregnancies 0 (%) | 33 (27.0) | 19 (32.8) | 23637 (38.8) | 0.43 | <0.01 | 0.29 |
| Former pregnancies 1 (%) | 42 (34.4) | 21 (36.2) | 19395 (31.9) |  |  |  |
| Former pregnancies 2+ (%) | 47 (38.5) | 18 (31.0) | 17839 (29.3) |  |  |  |
| Gestational age at delivery (SD) | 280 (7.70) | 270 (11.1) | NA | <0.001 |  |  |
| Infant sex Boy (%) | 63 (51.6) | 33 (58.9) | 31166 (51.2) | 0.36 | 0.90 | 0.21 |
| Infant sex Girl (%) | 59 (48.4) | 23 (41,1) | 29705 (48.8) |  |  |  |
| Infant weight, g mean (SD) | 3592 (457) | 3470 (567) | 3474 (609) | 0.18 | 0.04 | 0.95 |
| Apgar <7 at 5 min, n (%) | 0 (0.0) | 0 (0.0) | NA |  |  |  |
|  |  |  |  |  |  |  |
